# Alternative splicing: an important mechanism for myometrial gene regulation that can be manipulated to target specific genes associated with preterm labour

**DOI:** 10.1186/1471-2393-7-S1-S13

**Published:** 2007-06-01

**Authors:** Alison Jane Tyson-Capper

**Affiliations:** 1 Surgical and Reproductive Sciences, Institute of Cellular Medicine, Faculty of Medicine, Newcastle University, Framlington Place, Newcastle upon Tyne, NE2 4HH, UK

## Abstract

Considerable effort has been expended in attempting to distinguish genes that contribute to initiating the onset of term and preterm labour (PTL) from those that change in expression as a consequence of the progression of labour. The ability to define more clearly the genes involved in triggering labour contractions should lead to the development of new effective and safer strategies to prevent preterm birth. There is ample evidence to suggest that specific genes are co-ordinately regulated within the upper and lower regions of the myometrium prior to and during parturition and many of these genes are regulated by alternative pre-mRNA splicing. This mini-review highlights that expression of a range of different splicing factors, with defined roles in pre-mRNA splicing, is both temporally and spatially regulated within the uterine smooth muscle during pregnancy and labour. Moreover, several of these splicing factors play key roles in controlling the differential expression of specific regulatory proteins involved in uterine signalling and uterine quiescence. In addition, antisense morpholino oligonucleotide manipulation of pre-mRNA splicing may have potential in defining and targeting uterine pro-labour genes and thus contribute to the development of new therapeutic approaches to prevent PTL.

## Background

### Splicing factors and myometrial genes

**Figure 1:**
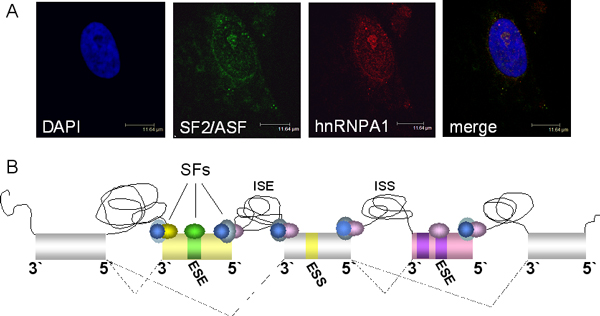
**Factors involved in alternative pre-mRNA splicing**. **(A) **Co-localisation of the antagonistic splicing factors SF2/ASF and hnRNPA1 within the nuclei of human myometrial cells by fluorescence confocal microscopy; Nuclei (blue) were defined by staining with 4',6-diamidino-2-phenylindole (DAPI) which binds to double stranded DNA; Co-localisation of SF2/ASF (green fluorescence) and hnRNPA1 (red fluorescence) was observed using monoclonal antibodies specific to each protein. **(B)** Regulation of alternative pre-mRNA splicing involves many different nuclear *trans*-acting splicing factors (SF), such as SR protein members, e.g SF2/ASF and hnRNPs, e.g hnRNPA1 and snRNPs that recognise and interact with numerous *cis*-acting RNA motifs present within the pre-mRNA sequence of genes; these include 5'donor and 3'acceptor sites at exon: intron boundaries, exon and intron enhancer elements (ESE, ISE) which can promote the use of specific splice sites; exon and intron silencer elements (ESS, ISS) which can when bound by hnRNPs repress the use of specific splice sites. Coloured boxes represent alternatively spliced exons.

**Figure 2:**
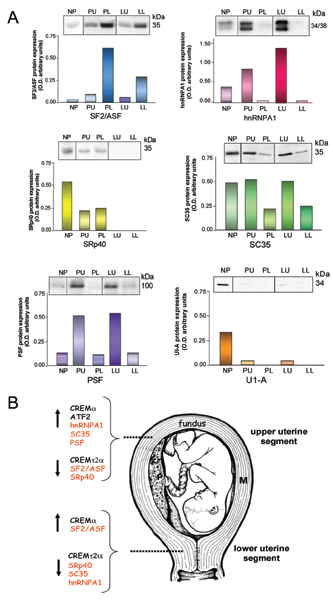
**Spatial and temporal expression of splicing factors in the myometrium during pregnancy**. **(A) **SF2/ASF, hnRNPA1, SRp40, SC35, PSF and UI-A snRNP protein expression within the upper and lower uterine regions of the human myometrium. Data are shown as the mean (*n *= 10) for non-pregnant (NP), term not in labour; upper (PU) and lower uterine regions and labouring upper (LU) and lower uterine regions (LL). **(B) **The switch in the expression of SF2/ASF, hnRNPA1, SRp40, SC35, PSF and UI-A snRNP within the upper and lower uterine regions from the non-pregnant to the term pregnant is indicated (red). Differential expression profiles for spliced variants of the transcriptional factors CREMτ_2_α, CREMα and ATF-2 are also shown (black).

**Figure 3:**
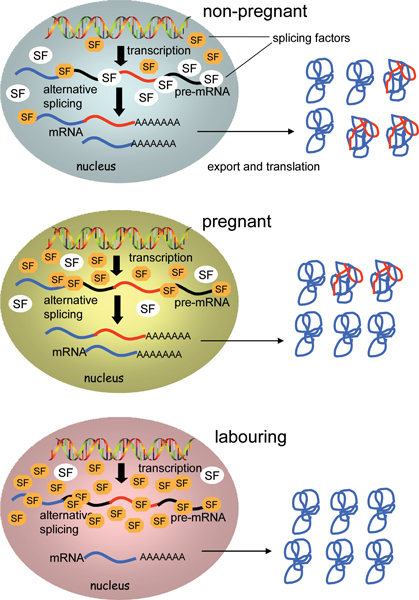
**Cartoon indicating the effect that changes in expression of splicing factors can have on the expression myometrial genes**. Fluctuations in the levels, ratios and activity of myometrial nuclear splicing factors in pregnancy may influence the expression of substrate-specific pre-mRNAs and in turn influence and switch the expression and ratios of the specific protein isoforms that are generated. In theory, changes in the levels of splicing factors could generate a) proteins with enhanced activity; b) proteins with different functions; c) proteins with abolished functions and d) provide a means to regulate gene expression by increasing the rate of nonsense-mediated decay of affected mRNAs.

Alternative pre-mRNA splicing plays a major role in regulating gene expression and generating protein diversity and this mode of regulation appears to be particularly prevalent in smooth muscle myogenesis and contractility [1]. Myometrium is a unique smooth muscle organ that differentiates during pregnancy into functionally distinct upper and lower uterine regions. The myometrial processes regulating the activity of the uterus during gestation and parturition are associated, in part, to the differential expression and function of specific genes including: cyclooxygenase ll (COX-2), oxytocin receptors, progesterone receptors, specific prostaglandin receptors, corticotrophin-releasing hormone receptors (type 1), GTP-binding proteins (Gαs) [2-5]. Most of these latter examples exist as distinct protein isoforms generated from alternate pre-mRNA splicing. Alternative splicing is a fundamental and tightly regulated RNA processing event that involves a complex interplay of many different *trans*-acting splicing factors and numerous *cis*-acting regulatory elements present within the precursor mRNA sequences of eukaryotic genes (Figure 1) [6]. Some of these splicing regulatory proteins including SF2/ASF, hnRNPA1, SRp40, SC35, PSF and U1-A snRNP are spatio-temporally regulated within the upper and lower regions of the myometrium during pregnancy (Figure 2) [7]. SF2/ASF and hnRNPA1 have been studied extensively and have an antagonistic relationship in which changes in the nuclear concentration, ratio or activities of either protein can switch the expression of specific isoforms [8] as illustrated in Figure 3. In light of this it is not surprising that the polarisation of SF2/ASF and hnRNPA1 proteins that exists within the myometrium during pregnancy modulates the expression of alternate spliced myometrial proteins. Evidence indicates that both SF2/ASF and hnRNPA1 contribute to the up-regulation of the myometrial Gαs spliced variants that occurs during pregnancy [9]. Up-regulation of Gαs isoforms together with Gαs coupled adenylyl cyclase activity results in elevated cAMP levels [10], which has been proposed to be important for the maintenance of myometrial quiescence during gestation [11]. Interestingly, other components of the cAMP signalling pathway also undergo distinct patterns of alternate splicing within the myometrium during pregnancy; examples include adenylyl cyclase isoforms [12], the cAMP-dependent transcription factors, cAMP-response element modulator proteins, CREM (CREMτ_2_α and CREMα) and the activating transcription factor 2 (ATF-2) [13]. Alternative splicing of CREM promotes a 'switch' within the myometrium during pregnancy and labour from production of CREMτ_2_α, a potent transcriptional activator to the synthesis of CREMα, a transcriptional repressor. These changes in splicing patterns are primarily controlled by the differential expression and activity of the splicing factor, SRp40 (Figure 2) and multiple SRp40 binding RNA motifs that are present within the alternatively spliced exons of CREM [14]. Since there is ample evidence for the physiological relevance of CREM mediated gene regulation, these changes in the expression of functionally distinct CREM spliced variants are likely to have biological consequences on the expression of down-stream target genes involve in uterine activity during gestation and parturition.

### Antisense oligonucleotides as modulators of myometrial alternative splicing

**Figure 4:**
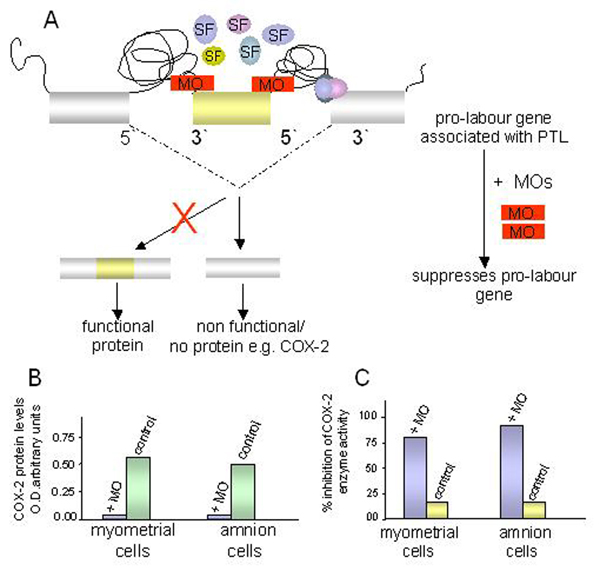
**Targeting pro-labour genes with antisense morpholino oligonucleotides**. **(A) **Diagram to show mechanism of action of morpholino oligonucleotides (MOs) in targeting nuclear pre-mRNA. Binding of (MOs) to the 5' and 3' splice site boundaries blocks access by splicing factors (SF) to the splice sites and redirects the splicing machinery to skip out the exon. This results in a shortened mRNA transcript and a truncated protein or no protein. **(B) **Effect of MOs targeted to the pre-mRNA of exon 4 of the COX-2 gene. Substantial inhibition by MOs of COX-2 protein expression, induced by LPS, was observed at the protein level, means ± S.E.M. (*n *= 6). **(C) **Loss of enzyme activity due to MOs inhibition was also determined; this assay measures COX-2 activity by calculating the rate of conversion of arachidonic acid to PGH_2 _[18]. Data shown as % inhibition. Data are mean ± S.E.M. (*n *= 6).

The use of antisense oligonucleotides to manipulate the expression of genes *in vitro *is well established [15,16]. Various chemically modified antisense systems have been used against several 'disease-causing genes', such as the dystrophin gene and the survival of motor neuron gene (SMN2), to manipulate the consequences of mutations present at splice sites that would normally generate an aberrant or truncated protein [16]. The ability to modify the alternative splicing pattern(s) of genes by targeting pre-mRNA with antisense oligonucleotides has also provided a useful experimental strategy to elucidate the function of different ratios of spliced variants and a potential therapeutic strategy to increase/decrease the ratio of specific spliced variants known to be associated with certain cancers and other diseases [16,17]. A similar approach has been adopted to use antisense oligonucleotides to switch splice site selection and redirect the splicing machinery to skip out exons that encode for either a functional domain or induces a premature stop codon. In principal this generates a protein devoid of functional domains or no protein at all (Figure 4A). A similar strategy could therefore be utilised to define and suppress the activation of specific genes that may trigger the onset of term and preterm labour. COX-2 is one such gene that has been implicated in contributing to the onset of both term and preterm labour. It has recently been shown that morpholino oligonucleotides targeted to both the 5' donor and 3'acceptor splice site boundaries of the exon 4 of COX-2 pre-mRNA significantly inhibited the expression and activity of this enzyme (induced by lipopolysaccharide) in both myometrial and amnion-derived cultured cell monolayers [18]. Morpholino antisense oligonucleotides have a number of advantages over other gene silencing systems; they are DNA analogues so are resistant to enzymatic degradation; they have very low toxicity; long term activity; excellent target specificity compared to alternative gene-silencing systems; consequently they represent a highly specific and non-toxic technique to define the function and silence specific genes.

## Conclusion

To summarise, alternative splicing plays an important role in modulating myometrial gene regulation during pregnancy and labour by naturally increasing the coding capacity of myometrial mRNA transcripts to generate different protein isoforms with enhanced activity and also isoforms with markedly different activities. This mini-review proposes that alternative splicing within the myometrium during pregnancy, term and preterm labour is not likely to be a static process but subject to change. The expression profiles for splicing factors change dramatically within the myometrium in pregnancy; these factors have been shown to have substrate-specific roles in alternative splicing and it is possible that they may modulate the expression of many more alternately spliced myometrial proteins involved in myometrial quiescence and contractility. Therefore, a more complete analysis of alternative splicing events coupled with transcriptional control of myometrial genes would be advantageous to increase our understanding of the complex co-ordinated molecular processes that regulate myometrial genes in pregnancy and labour. Finally, the use of highly specific antisense morpholino oligonucleotides may prove to be a valuable technique to identify and 'switch off' specific genes the activation of which is linked with the initiation of term and preterm labour.

## Competing interests

The author declares that they have no competing interests.
